# Schwann cell precursors as a source for adrenal gland chromaffin cells

**DOI:** 10.1038/cddis.2017.456

**Published:** 2017-10-05

**Authors:** Luiza Lousado, Pedro H D M Prazeres, Julia P Andreotti, Ana E Paiva, Patrick O Azevedo, Gabryella S P Santos, Renato Filev, Akiva Mintz, Alexander Birbrair

**Affiliations:** 1 Department of Pathology, Federal University of Minas Gerais, Belo Horizonte, MG, Brazil; 2 Laboratory of Neurobiology, Department of Neurobiology, Federal University of São Paulo, SP, Brazil; 3 Department of Radiology, Columbia University Medical Center, New York, NY, USA; 4 Department of Cell Biology, Albert Einstein College of Medicine, Bronx, NY, USA; 5 Ruth L. and David S. Gottesman Institute for Stem Cell and Regenerative Medicine Research, Albert Einstein College of Medicine, Bronx, NY, USA

Schwann cells are cells defined by their intimate relationship with axons in the peripheral nervous system throughout development.^1^ Schwann cell precursors depict the earliest developmental stage of the Schwann cell lineage.^2^ Schwann cell precursors arise from migrating neural crest cells.^3^ During embryonic development, Schwann cell precursors migrate along peripheral neuronal axons to their final destinations.^4^ Compared to mature Schwann cells, Schwann cell precursors have an increased survival and migratory capacity.^5^ Although Schwann cell precursors were historically considered primed toward Schwann cell differentiation, as their name states; recent studies suggest that these cells may behave as progenitors of other cell types as well. Schwann cell precursors give rise to parasympathetic^4, 5^ and enteric neurons,^2^ melanocytes,^3^ endoneural fibroblasts, and odontoblasts.^6^ As the Schwann cell precursors escort growing nerves to nearly every organ during development, it is possible that their role as progenitors was downplayed in some unstudied tissues. Thus, whether Schwann cell precursors can differentiate into other cell types remains elusive.

The adrenal gland contains secretory neuroendocrine cells in its medullar region, which are named chromaffin cells due to their production of colored polymers of catecholamines after exposure to the oxidizing agent chromate.^7^ Morphologically, chromaffin cells resemble endocrine cells by their lack of neurites, and large storage vesicles, with chromaffin granules.^7^ These cells synthesize and store hormones, peptides, and small molecules, which are secreted into the blood circulation, playing crucial roles in numerous physiological conditions, that is, vascular perfusion.^7^ Despite the importance of chromaffin cells, few studies have been done to reveal their exact origin. Understanding the origin and the processes that drive the formation of chromaffin cells is a central question in developmental biology.

Early developmental studies introduced the idea and the general consensus holds that both adrenal chromaffin cells and sympathetic neurons are derivatives of the same sympathoadrenal progenitor. Now, in a recent study in *Science*, Adameyko’s group challenge the current view about chromaffin cells’ origin by using state-of-the-art techniques, including sophisticated *in vivo* inducible genetic lineage-tracing approaches, specific Schwann cell precursor depletion, and genetic denervation.^8^ Their data revealed that during development Schwann cell precursors are the ancestors of adrenal medullar chromaffin cells.^8^ The authors investigated the progeny of Schwann cell precursors by using Plp1-CreERT2/R26R^YFP^ mice to track specifically Schwann cell precursor-generated cells. These experiments unveiled that approximately half of chromaffin cells are derived from nerve-associated Schwann cell precursors.^8^ Furthermore, Furlan and colleagues^8^ showed defective chromaffin cell production in the adrenal medulla in Sox10-CreERT2/R26R^DTA^ mice, in which Schwann cell precursor ablation was induced at E11.5, indicating the necessity of Schwann cell precursors for chromaffin cell formation. In addition, the authors analyzed denervated adrenal medulla in HB9-Cre/Isl2DTA mice. Strikingly, the denervation caused a reduction in the number of chromaffin cells, adding evidence that Schwann cell precursors, which are attached to the nerves, are essential for chromaffin cell generation.^8^ Moreover, this study examined mice deficient in a critical gene for chromaffin cell differentiation (Ascl1 knockout mice). Furlan and colleagues^8^ show that inhibiting chromaffin cell differentiation, leads to accumulation of Schwann cell precursors that fail to differentiate.

Although it has long been known that Schwann cell precursors have the capacity to differentiate into mature Schwann cells, the findings by Furlan *et al.*^8^ suggest that Schwann cell precursors are equally crucial for adrenal gland formation outside of the nervous system. The impressive unexpected plasticity of Schwann cell precursors indicates that these cells possibly affect tissue regeneration more broadly that previously was thought. This remarkable capacity of Schwann cell precursors opens the door to hypothesis about their unexplored roles in other organs, and represents a promising tool and research direction in regenerative biology. Here, we discuss the findings from this work, and evaluate recent advances in our understanding of the adrenal medulla biology.

## Perspectives/future directions

**Figure 1:**
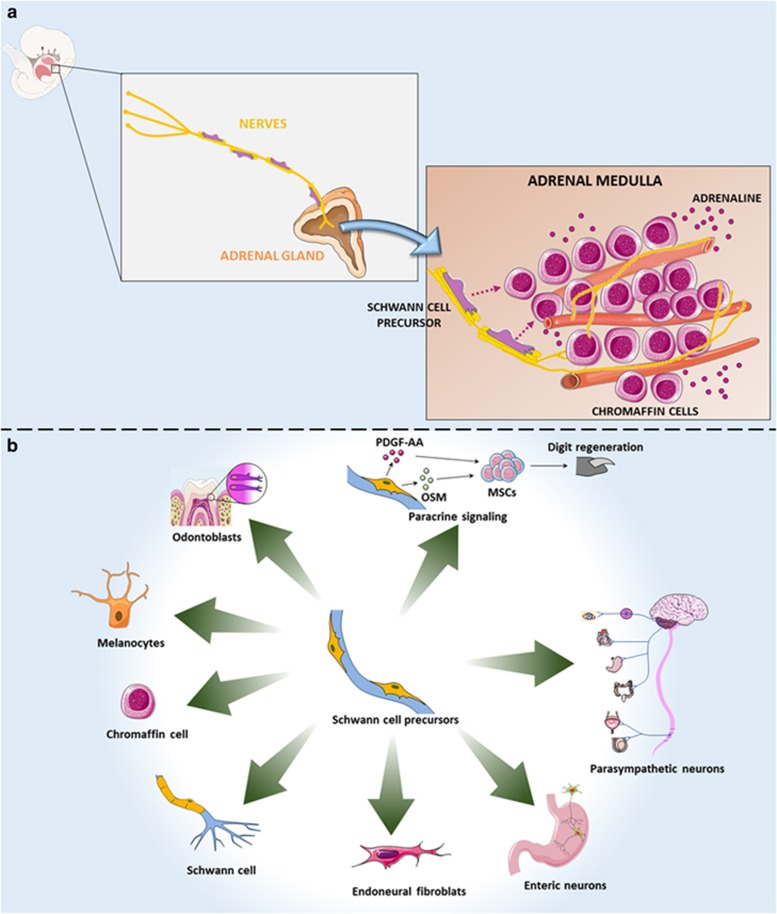
Adrenal chromaffin cells are derived from Schwann cell precursors during embryonic development. (**a**) The adrenal medulla contains secretory chromaffin cells which synthesize and store hormones crucial for numerous physiological conditions. Understanding the origin and the cellular mechanisms that drive the formation of chromaffin cells is a central question in developmental biology. Furlan and colleagues^8^ now show that Schwann cell precursors generate adrenal medullar chromaffin cells. Future studies may reveal the complexity of the adrenal medulla microenvironment important for chromaffin cells formation in much greater detail. (**b**) Schwann cell precursors as multipotent stem cells. Schwann cell precursors line axons, and migrate long distances following them. Recent studies have shown that under specific conditions these cells can detach from the nerve projections, and generate, in addition to mature Schwann cells, odontoblasts,^6^ melanocytes,^3^ chromaffin cells,^8^ endoneural fibroblasts, enteric,^2^ and parasympathetic^4, 5^ neurons. Moreover, Schwann cell precursors also can regulate the functioning of other stem cells, by paracrine secretion.^15^

Myelinating and nonmyelinating Schwann cells, melanoblasts,^3^ odontoblasts,^6^ and now also chromaffin cells^8^ all derive from Schwann cell precursors associated with peripheral axons during embryonic development. This brings the question whether the same Schwann cell precursor is capable of differentiating into these variable cell types. Are Schwann cell precursors multipotent stem cells? Schwann cell precursors line nerve projections, facilitate their survival, and are able to migrate long distances following them. It remains unknown whether Schwann cell precursors associated with distinct neuronal types also present differences in their differentiation capabilities. The heterogeneity of Schwann cell precursors was not yet defined. Thus, it remains to be elucidated whether they correspond to a homogeneous cellular population or not. Future studies should reveal whether a particular Schwann cell precursor phenotype relates to a precise differentiation capacity, and whether Schwann cell precursor oligopotent or unipotent subpopulations exist already pre-committed to specific lineages (Figure 1).

The decisions of progenitors to self-renew or differentiate are dependent on the interaction of those with their surrounding microenvironments, also called ‘niches’,^9^ in different organs.^1, 10, 11^ Thus, pro-quiescence, pro-differentiation or pro-self-renewal niches define the progenitor’s fate.^1^ The deregulation of those niche regulatory mechanisms plays a key pathogenic role in a variety of disorders.^1^ In recent years, several cell types have been identified as potential niche-supporting cells for progenitors in other organs, including endothelial cells, pericytes, hematopoietic cells, smooth muscle cells, and others.^1, 12, 13^ What is the composition of adrenal medulla niche which supports the formation of chromaffin cells? What are the cellular and molecular regulatory mechanisms involved in this process? Thus, future experiments should be designed to address what are the molecular mechanisms that guide Schwann cell precursors lining nerves to detach and differentiate into chromaffin cells.

The adrenal medulla contains at least two types of chromaffin cells, adrenaline- and noradrenaline-producing cells.^7^ Interestingly, it seems that not all chromaffin cells are derived from nerve-associated Schwann cell precursors. Furlan and colleagues suggest that <50% of these cells are derived from Schwann cell precursors, as demonstrated by traced chromaffin cells in Plp1-CreERT2/R26R^YFP^ mice.^8^ However, it remains to be revealed which are the other sources of chromaffin cells in the adrenal gland. The data from this study suggest that chromaffin cells are heterogeneous with regard to their origin. Future studies should explore whether the subset of chromaffin cells derived from Schwann cell precursors differ from the other chromaffin cells also in function (for example, in the production of adrenaline, noradrenaline, chromogranin, and neuropeptides) and in other characteristics.

Regarding the adrenal gland innervation, it is generally accepted that the organ is innervated by various nerve fibers of both intrinsic and extrinsic origin.^7^ These innervations have been reported to participate in the control of several functions of the adrenal gland.^7^ Furlan and colleagues showed that denervation affects chromaffin cell differentiation. Nonetheless, is there a subgroup of nerves that is more important? Do the Schwann cell precursors from different nerves differ in their differentiation capacity in the adrenal gland? The genetic denervation caused a decrease of 78% in the total number of chromaffin cells in the adrenal medulla.^8^ The authors suggested that this reduction was possibly due to the ablation of Schwann cell precursors, as those cells migrate along the nerves. Nevertheless, as only half of chromaffin cells are originated from Schwann cell precursors, the nerves themselves may be important for chromaffin cells formation. Signals from the peripheral nervous system have been identified as a regulatory component of the stem cell niche in several organs.^1^ The nerves may produce peptides which are delivered to the tissue microenvironment by secretion from the nerves endings acting by paracrine signaling and affecting other cell types.^1^ Future studies should reveal whether the nerves themselves independent of Schwann cell precursors are important for chromaffin cells formation. To study specifically the role of particular innervations in chromaffin cell differentiation, nerve-specific inducible genetic approaches should be used in combination with DREADDs, which can modulate neural activity, to inhibit nerve projections activity without killing the nerves themselves. Thus, affecting only the nerves, but not the Schwann cell precursors attached to them.

Chromaffin are a type of neuroendocrine cells, which receive neuronal input and, as a consequence, release message molecules.^7^ The neuroendocrine cells are also found in several other organs besides the adrenal gland, including lungs, thyroid, gut, pancreas, ovaries, and prostate. The embryonic origin of those cells is not well understood, and in some cases completely unknown. Deciphering the origins and the processes that drive the formation of neuroendocrine cells will be important in advancing our understanding of development and disease in those organs. Interestingly, the present finding of chromaffin cells origin raises the possibility that Schwann cell precursors may generate other neuroendocrine cells as well. Thus, further studies will reveal the origin of neuroendocrine cells in other tissues.

Importantly, Furlan and colleagues^8^ show that chromaffin cells differentiate from Schwann cell precursors in the embryonic stage. Can this process also occur during adult life? Removal of adrenal cortex and medulla triggers rapid gland growth, leading within relatively short time to the restoration of adrenal structure and function.^14^ Is this regeneration in the adult life also dependent of cells from the Schwann cell lineage? A fascinating aspect of Schwann cells derived from Schwann cell precursors is their ability to dedifferentiate and re-enter the cell cycle in response to tissue damage. Are the chromaffin cells able to dedifferentiate after adrenal injury as well? In addition, a recent study has shown that Schwann cell precursors secrete paracrine factors that control the behavior of other progenitors.^15^ Does the role of Schwann cell precursors in chromaffin cell formation also involves secretion of important molecules for the differentiation of those cells?

In conclusion, understanding the origin and the cellular processes involved in chromaffin cell generation in the adrenal medulla is a central question in developmental biology. What is the ancestry of all chromaffin cells remains unknown. Furlan and colleagues^8^ reveal a source for half of chromaffin cell population: Schwann cell precursors. This new knowledge advances our comprehension of chromaffin cell biology. This remarkable capacity of Schwann cell precursors represents promising potential tool and research direction for therapeutic application of these cells outside of the nervous system.
